# A Transition Course to Reverse Empathy Decline in Medical Students

**DOI:** 10.1111/tct.70138

**Published:** 2025-07-03

**Authors:** Jeremy Howick, Daniel Slavin, Amber Bennett‐Weston, Catherine Eyres, Andy Ward, Leila Keshtkar, Sophie Parkinson, Chris Williams, Josie Solomon‐Taylor

**Affiliations:** ^1^ Stoneygate Centre for Excellence in Empathic Healthcare, Leicester Medical School, College of Life Sciences, George Davies Centre University of Leicester Leicester UK; ^2^ Leicester Medical School, College of Life Sciences, George Davies Centre University of Leicester Leicester UK; ^3^ School of Allied Health Professions, Nursing and Midwifery Sheffield University Sheffield UK

**Keywords:** empathy, medical students, transition course

## Abstract

**Background:**

Medical students report rising stress and lowering empathy as they progress from the pre‐clinical to the clinical phase of training. Transition courses can attenuate or even reverse this. We developed and evaluated a transition course at Leicester Medical School.

**Approach:**

The transition course included: (1) near‐peer mentoring with third‐ and fifth‐year medical students; (2) role model training for clinical tutors; (3) a tutorial for third‐year students on identifying positive role models and (4) an ‘empathy champion’ scheme. The first and second components were evaluated with satisfaction surveys and interviews, the third with a satisfaction questionnaire and the fourth by counting nominated empathy champions.

**Evaluation:**

Twenty‐four pairs of third‐ and fifth‐year medical students participated in the near‐peer scheme, with 18 students participating in interviews. Role model training was delivered to over 500 healthcare professionals; 295 survey respondents reported an average confidence of 8.4 out of 10 in their ability to implement their learning. Ninety‐six students attended the tutorial on identifying positive role models; almost all (93%) agreed that it was useful. Thirty‐seven empathy champions have been nominated.

**Implications:**

Our transition course was well received and addresses complex issues that may contribute to a decline in medical student empathy at a critical training juncture. Educators should ensure that transition courses to promote empathy consider the increasing complexity of patients, stressful culture and lack of positive role models that students encounter upon entering the clinical phase of training. Future research should identify ways to optimise, expand and evaluate the course more extensively.

## 1 Background

Clinical empathy involves exploring and understanding the patients' perspective, reaching a shared understanding with the patient, feeling in response to understanding and taking therapeutic action, while maintaining boundaries (see Table 1 for a description of each component of clinical empathy) [1]. For patients, clinical empathy improves outcomes ranging from pain to quality of life [2, 3, 4], while also enhancing practitioner wellbeing and job satisfaction [5, 6]. Despite these benefits, medical student empathy often declines throughout medical school [7]. This decline appears to be more acute as students transition from the pre‐clinical to the clinical phase of training [8].

**TABLE 1:** A summary of the components of clinical empathy [1].

Component of clinical empathy	Description
**Exploring**	Involves the practitioner actively seeking information about, and actively listening to, the patient's perspective. It includes having curiosity about the patient's wider social, cultural, religious, moral, and familial background, and should be non‐judgemental.
**Understanding**	Involves the practitioner developing their own understanding of the patient's perspective through cognitive processes, including using their imagination, logic, and reasoning to put themselves in the patient's shoes.
**Shared understanding**	Involves the practitioner and patient, together, reaching a shared understanding of the patient's circumstance, values, preferences, and options. This is achieved by the practitioner communicating their own understanding back to the patient to check its accuracy.
**Feeling**	Involves feeling something in response to the patients' circumstance (e.g., of compassion or concern). This is not about the practitioner feeling the same emotions as the patient.
**Therapeutic action**	The caring behaviours performed by practitioners to help their patients, for example, establishing a care management plan or a course of treatment. This can also involve simply showing the patient that they have listened to them or understood their perspective.
**Maintaining boundaries**	Involves the practitioner actively maintaining a distinction between the ‘self’ and the ‘other’ such that the practitioner's own emotions, experiences, and expectations are recognised as separate from the patient's.

*… medical student empathy often declines … as students transition from the pre‐clinical to the clinical phase of training*.

This trend has been attributed to two main factors. Firstly, medical students on placement are faced with more complex patients than most have been exposed to in their previous lecture theatre or personal experience. Secondly, they are exposed to a ‘hidden curriculum’ which includes a stressful organisational culture, little formal empathy teaching, prioritisation of biomedical knowledge and paucity of positive role models [9].

While overcoming these challenges can be complex [10, 11, 12], fortunately, empathy can be taught [13], implying that the empathy decline could be reversible. Transition courses that help students adapt to their placements can achieve this [9]. A scoping review of transition courses recommended that they include educational (problem‐based learning or early patient contact), social (introduction to staff or near‐peer relationships) and developmental (student reflection) components [14]. Transition courses can improve student performance [15]. However, previous research has not explored whether they can reverse empathy decline [14].

Here, we describe the implementation and evaluation of a transition course at Leicester Medical School, which was designed to address educational, social and developmental components [14].

## 2 Approach

### 2.1 Context

An audit of the Leicester Medical School curriculum (unpublished) revealed the absence of a formal near‐peer programme and of interventions to offset the empathy draining effect of un‐empathic role models. To address this, our transition course included four elements: (1) near‐peer mentoring with pairs of third‐ and fifth‐year medical students, (2) role model training for clinical tutors at placement sites, (3) tutorial for third‐year students on identifying positive role models and (4) an ‘empathy champion’ scheme to encourage potential role models to be more empathic.

### 2.2 Near‐Peer Mentoring

Mentoring helps students to learn timely, clinically relevant knowledge, and to identify gaps in their learning or wellbeing that the university may be unable to address [16]. It also helps mentors acquire organisational, interpersonal and teaching skills [16]. We used convenience sampling to recruit all third‐year mentees and fifth‐year mentors to participate in a near‐peer mentoring programme. At the end of the programme, students reflected on their learnings and future applications. The programme was evaluated using semi‐structured interviews, which were analysed by four authors using reflexive thematic analysis [17]. The programme first took place during the 2022–2023 academic year, and a full report of this component of the transition course and its evaluation is available elsewhere [18].

### 2.3 Role Model Training for Clinical Staff

Role modelling is key to professional development, including the development of empathy [9]. To enhance the empathy of clinicians that students were likely to encounter, a series of empathy workshops were conducted by educators from the Stoneygate Centre for Empathic Healthcare in Leicester, Northamptonshire, Rutland, and Hampshire during the 2023/2024 and 2024/2025 academic years (Table 2). The sessions were evaluated through questionnaires that assessed participant satisfaction and confidence in their ability to implement their learning.

**TABLE 2:** Empathy training of healthcare professionals conducted at time of submission.

Date	Training	Participants	Number of participants
14/09/2023	Teaching Empathy in Primary Care	GP Educators who teach Leicester Medical Students	12
28/09/2023	System Empathy Training for Healthcare Practitioners	Primary Care staff (clinical and non‐clinical) from a local Primary Care Network	59
29/09/2023 and 12/10/2023	Empathy in Teams for Healthcare Practitioners (2 x one day course)	Nurses, Midwives and Allied Health Professionals (AHPs) from University Hospitals Leicester (UHL)	87
10/10/2023	System Empathy Training for Healthcare Practitioners	All grades of staff (clinical and non‐clinical) from Emergency Department, UHL	28
02/11/2023	Putting evidence‐based empathy into practice (half day)	Post‐graduate doctors in training (on GP training scheme)	46
07/11/2023	System Empathy Training for Healthcare Practitioners	Nurses, Midwives and AHPs from UHL	40
30/07/2024 and 01/08/2024 (online)	Putting evidence‐based empathy for teams into practice	Clinical and Non‐Clinical Staff from UHL Maternity Services	41
22/08/2024 (online) and 19/09/2024	Putting evidence‐based empathy into practice	Clinical and Non‐Clinical Staff from UHL Maternity Services	70
20/09/2024	System Empathy Training	Clinical and Non‐Clinical Staff from UHL Maternity Services	31
08/10/2024	Putting evidence‐based empathy into practice	Clinical and non‐clinical staff from primary, secondary and tertiary care. Medical educators.	29
16/10/2024	Teaching Empathy in Clinical Settings	Senior clinical educators and leaders from primary and secondary care in the East Midlands	40
05/11/2024	Putting evidence‐based empathy for teams into practice	Clinical pathologists, bio‐medical scientists, phlebotomists, leadership team in a Pathology Department	20
**Total**			**503**

### 2.4 Tutorial on Identifying Positive Role Models

The role model training did not reach all clinicians that students would encounter, meaning students would likely face, and potentially be influenced by, some un‐empathic role models. To address this, a 90‐min tutorial was developed and delivered in 2022 to help the students consciously identify (aspects of) empathic role models. The tutorial design was refined to provide students with opportunities to apply their theoretical learning to their own practice, based on a co‐production workshop that included student input. The tutorial used the model‐trialling cycle [19] to help students adopt positive behaviours by reflecting on aspects of behaviour they deemed to be congruent with an empathic or un‐empathic role model. During this cycle, the student attempts to incorporate a trait or behaviour into their own professional practice and test its worth. Students completed a feedback survey containing four items (Table 3) followed by two free‐text questions about their experiences of the tutorial.

**TABLE 3:** Summary of results from the survey after the positive role modelling training.

Question	Agree	Strongly agree
This topic is relevant to me as a medical student	48.1%	48.1%
This session has helped me to identify my current role models in medicine	63%	29.6%
This session will help me identify role models in the future	46.3%	44.4%
I will be able to apply this during my time in the clinical setting	44.4%	40.7%
Do you think that this session will reduce the chances that your empathy will decline?	37%	14.8%

### 2.5 ‘Empathy Champion’ Scheme

To further encourage potential role models to enhance their empathy, an ‘empathy champion’ scheme was developed and implemented. A flyer explaining the scheme was sent to students and course tutors within Leicester Medical School in October 2024, inviting them to nominate peers or clinical tutors. Those receiving at least two nominations were awarded an ‘empathy champion’ badge, certificate and invited to a celebration which was shared on social media if they consented. The scheme was evaluated by recording the number of nominations and how many ‘empathy champions’ consented to have their award shared on social media.

### 2.6 Ethics

Ethical approval to evaluate the near‐peer programme (reference: 38089‐ds729‐ls: medicine) and the role model training for clinical staff (reference: 2024‐1125‐1269) was granted by the University of Leicester Research Ethics Committee. The remaining programmes were delivered as part of routine teaching and ethical approval was not required.

## 3 Evaluation

### 3.1 Near‐Peer Mentoring

Twenty‐four pairs of students participated in the scheme, and eight third‐year mentees and 10 fifth‐year mentors participated in interviews between May and July 2023. All participants volunteered to take part in the programme following an email invitation. Four themes captured students' views and experiences: the value of near‐toto‐peer understanding, tailored advice, enhanced empathy and greater structure to maximise the benefits of the programme; these themes are described in greater detail in the full report of this component of the transition course [18].

### 3.2 Role Model Training for Clinical Staff

Between September 2023 and November 2024, empathy training was delivered to 503 healthcare practitioners. Participants included GPs, nurses, midwives and obstetric consultants (Table 1). Two hundred ninety‐five participants completed feedback surveys; overall satisfaction with the training averaged 8.6 out of 10, with scores ranging from 7.5 to 9.6. Respondents reported an average confidence of 8.4 out of 10 in their ability to implement their learning, with scores ranging from 7.4 to 9.1. In the free text comments, participants called for the training to ‘be mandatory for everyone’ and claimed that it had ‘positively changed their mindset to give better care to patients’.

*Positively changed their mindset to give better care to patients*.

### 3.3 Tutorial on Identifying Positive Role Models

Fifty‐four the 96 students who attended the tutorial completed the survey. Almost all of those surveyed (96.2%) agreed that the topic of role models and how to identify them was relevant to them (Table 3). Over half (61.1%) of those surveyed had considered their role models before the intervention. Most (92.6%) of the students agreed or strongly agreed that the intervention helped them to identify empathic role models, and 90.7% stated that the tutorial would help them identify future empathic role models.

In the free text comments, students reported appreciating the quality of the educator delivering the tutorial, finding the subject interesting, and noting that they would apply their learning in future practice. Suggestions for improvement included clearer task instructions and using an online platform to allow students to contribute to the session anonymously. Students also recommended providing examples of good and bad mentors.

### 3.4 ‘Empathy Champion’ Scheme

At the time of writing, 37 ‘empathy champion’ awards have been given, with nominations highlighting compassion, support and understanding towards patients and colleagues. Five ‘empathy champions’ agreed to have their photos taken and to have their achievements posted on the Stoneygate Centre for Empathic Healthcare's social media.

## 4 Implications

### 4.1 Summary of Findings

A four‐pronged transition course was successfully delivered to medical students at the Leicester Medical School. Students, mentors and healthcare practitioners reported being satisfied with the course, and most reported it would reduce the chances that their empathy would decline.

*A four‐pronged transition course was successfully delivered to medical students at Leicester Medical School*.

### 4.2 Comparison With Other Evidence

A scoping review [14] found that most transition courses focus on educational interventions, neglecting social and developmental aspects. We overcame this limitation: our near‐peer mentoring programme supported learning on placement (educational) fostered student relationships (social) and encouraged reflective practice (developmental). Similarly, our role model training for clinical staff provided education about empathy (educational), built inter‐professional relationships (social) and prompted reflection on practice (developmental).

Previous studies have demonstrated the benefits of transition courses for improving academic performance, reducing stress and enhancing professional development [14, 15]. Our findings confirm this. For instance, the near‐peer programme provided students with support, while the tutorial on identifying role models helped students reflect on their professional identities.

Moreover, whereas previous evaluations of overall transition courses have not explored the effects on students' empathy levels, our evaluation found that students perceived that the course would improve their empathy [14].

*… our evaluation found that students perceived that the course would improve their empathy*.

### 4.3 Strengths and Limitations

Strengths of this report include that it brings together four related yet distinct elements of a transition course and that it included interventions for both medical students and role models [14]. There were also some limitations. First, we evaluated each component of the transition course separately, limiting the extent to which we can comment on its overall effect. Second, while students reported improved empathy, we did not measure this using a validated scale and did not assess whether patients found that their empathy increased. Third, the empathy champions were identified based on subjective reports. Finally, this course was delivered to medical students at one medical school, limiting the generalisability of the findings. However, our rich description of each component of the course supports reflection about its transferability to other contexts. Finally, the students in the near‐to‐peer programme volunteered to take part and may not have been representative.

### 4.4 Recommendations for Future Research and Practice

Further work is needed to expand the course in several ways, including making the near‐peer support available to all third‐year students at Leicester Medical School. Relatedly, a more extensive overall evaluation of the course is required to establish the extent to which the intervention is successful at reversing the decline in empathy. Finally, an exploration of the contextual factors that impact course effectiveness would be beneficial for other medical schools seeking to implement similar courses.

### 4.5 Conclusion

A transition course for medical students at the University of Leicester Medical School is feasible and rated highly by both students and role models. Future research is required to further evaluate the scheme, identify areas for development, and research contextual factors that will make it equally useful for other medical schools.

## Author Contributions

**Jeremy Howick:** conceptualization, investigation, writing – original draft, methodology, writing – review and editing, formal analysis. **Daniel Slavin:** methodology, writing – review and editing, writing – original draft, investigation, formal analysis. **Amber Bennett‐Weston:** writing – original draft, methodology, writing – review and editing, formal analysis. **Catherine Eyres:** writing – original draft, writing – review and editing, project administration, methodology. **Andy Ward:** writing – original draft, writing – review and editing, formal analysis, investigation. **Leila Keshtkar:** writing – original draft, methodology, formal analysis, writing – review and editing. **Sophie Parkinson:** writing – original draft, writing – review and editing, methodology. **Chris Williams:** writing – original draft, writing – review and editing, methodology. **Josie Solomon‐Taylor:** writing – original draft, methodology, formal analysis, writing – review and editing.

## Conflicts of Interest

The authors declare no conflicts of interest.

## Data Availability

The data that support the findings of this study are available from the corresponding author upon reasonable request.
